# Nimbolide Induces ROS-Regulated Apoptosis and Inhibits Cell Migration in Osteosarcoma

**DOI:** 10.3390/ijms161023405

**Published:** 2015-09-29

**Authors:** Ju-Fang Liu, Chun-Han Hou, Feng-Ling Lin, Ya-Ting Tsao, Sheng-Mou Hou

**Affiliations:** 1 Central Laboratory, Shin-Kong Wu Ho-Su Memorial Hospital, Taipei 11101, Taiwan; E-Mail: anti0822@hotmail.com; 2 Department of Orthopedic Surgery, National Taiwan University Hospital, Taipei 10617, Taiwan; E-Mails: chhou@ntu.edu.tw (C.-H.H.); m102094011@tmu.edu.tw (Y.-T.T.); 3 Department of Dermatology, Sijhih Cathay General Hospital, Taipei 22174, Taiwan; E-Mail: lavande213@gmail.com; 4 Department of Orthopedic Surgery, Shin-Kong Wu Ho-Su Memorial Hospital, No. 95, Wenchang Road, Shilin District, Taipei 11101, Taiwan

**Keywords:** osteosarcoma, apoptosis, endoplasmic reticulum stress, reactive oxygen species, migration

## Abstract

Osteosarcoma (OS) is a primary malignant tumor of bone and is most prevalent in children and adolescents. OS is frequently associated with pulmonary metastasis, which is the main cause of OS-related mortality. OS has a poor prognosis and is often unresponsive to conventional chemotherapy. In this study, we determined that Nimbolide, a novel anti-cancer therapy, acts by modulating multiple mechanisms in osteosarcoma cells. Nimbolide induces apoptosis by increasing endoplasmic reticulum (ER) stress, mitochondrial dysfunction, accumulation of reactive oxygen species (ROS), and finally, caspase activation. We also determined that Nimbolide inhibits cell migration, which is crucial for metastasis, by reducing the expression of integrin αvβ5. In addition, our results demonstrate that integrin αvβ5 expression is modulated by the PI3K/Akt and NF-κB signaling cascade. Nimbolide has potential as an anti-tumor drug given its multifunctional effects in OS. Collectively, these results help us to understand the mechanisms of action of Nimbolide and will aid in the development of effective therapies for OS.

## 1. Introduction

Osteosarcoma (OS) is the most common primary malignant bone tumor among children and adolescents. Surgery and systemic chemotherapy are the mainstay treatment for OS, and the cure rate is in the range of 58%–76% [1]. However, the survival rate has shown limited improvement. Today, the five-year survival rate is approximately 20% after surgical treatment alone. Chemotherapy is usually employed in an adjuvant situation to improve prognosis and long-term survival. However, recurrence usually manifests, sometimes as a local recurrence or metastasis to distant bones. More frequently, recurrence occurs as pulmonary metastasis, which results in a poor prognosis for many patients with OS [2]. Thus, a novel strategy that effectively inhibits metastasis, especially to the lung, from the primary site of OS is highly desirable.

**Figure 1:**
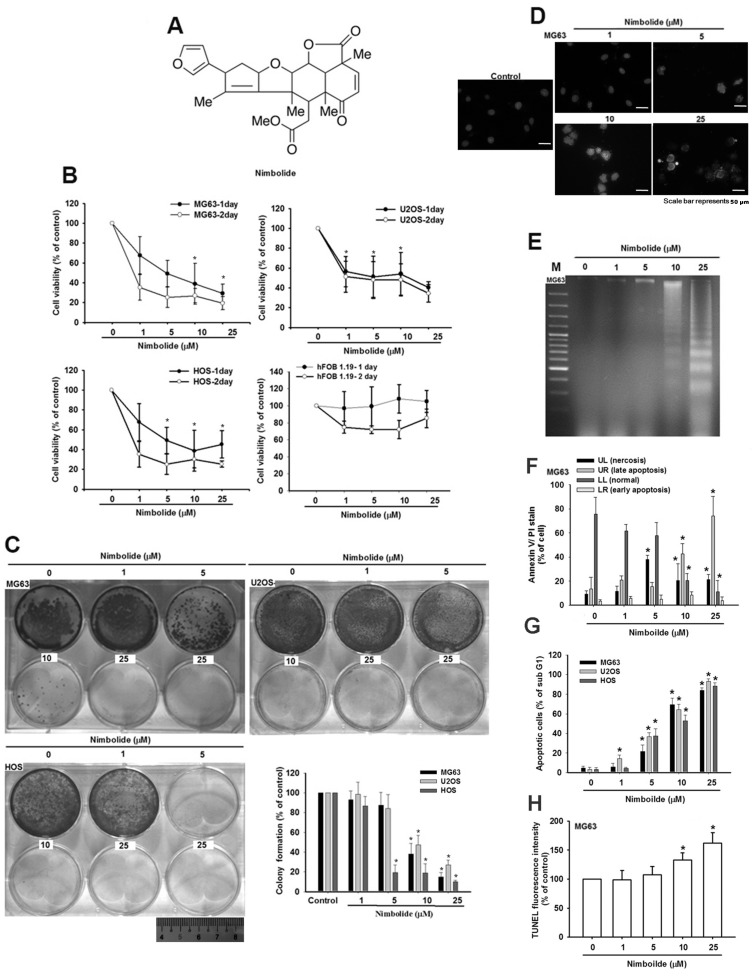
Nimbolide induces apoptosis in osteosarcoma cells. (**A**) The structure of Nimbolide. (**B**,**C**) Osteosarcoma cells (MG63, U2OS, and HOS) and osteoblast cells (hFOB 1.19) were incubated with control solution or various concentrations of Nimbolide for 1 or 2 days; (**B**) Cell viability was examined by using an MTT assay; (**C**) For the colony-formation assay, the cells were stained with crystal violet and photographed. The quantitative data are shown in the lower right panel; (**D**) MG63 osteosarcoma cells were incubated with control solution or various concentrations of Nimbolide for 24 h and nuclear morphology was recorded by using a DAPI stain and photography. Scale bar: 50 μm; (**E**) MG63 osteosarcoma cell lysates were collected for DNA purification, and the DNA was analyzed by gel electrophoresis; (**F**–**G**) Osteosarcoma cells were incubated with control solution or various concentrations of Nimbolide for 24 h, and the percentages of apoptotic cells were analyzed by flow cytometric analysis of (**F**) annexin V and PI double-labeling, (**G**) PI staining, and (**H**) TUNEL staining. The data are expressed as the mean ± SEM. * *p* *<* 0.05 as compared with the control group.

*Azadirachta indica* is a plant used in traditional medicine. Its extracts are reported to have anti-malarial [3] and anti-cancer properties [4,5]. Nimbolide, a constituent of *A. indica*, is a tetranortriterpenoid that consists of a classic limonoid skeleton with an α,β-unsaturated ketone system and a δ-lactone ring (Figure 1A). Previous studies have indicated that triterpenoids exhibit anti-proliferative activity against cancers such as breast cancer, lung cancer, neuroblastoma, OS, choriocarcinoma, and melanoma [6,7,8]. However, the mechanisms involved in the anti-tumor effects of Nimbolide have not been investigated.

In normal cells, reactive oxygen species (ROS) including superoxide anions (O_2_^−^), hydrogen peroxide (H_2_O_2_), and the highly reactive hydroxyl radical (·OH), are generated as by-products of cellular metabolism and are in cellular redox balance with biochemical antioxidants [9]. However, in many cancers, this vital balance is disrupted, resulting in oxidative stress and accumulation of ROS [10]. Because ROS can cause damage of DNA, RNA, and proteins, they contribute to the development of human diseases including insulin resistance, diabetes mellitus, and cancer [11,12,13]. In cancer cells, low levels of oxidative stress can promote survival and proliferation, but higher levels can induce apoptosis and cell cycle arrest [14,15,16]. The accumulated evidence indicates that apoptosis is associated with an increase in mitochondrial oxidative stress, release of cytochrome C, and the activation of caspases [17]. Traditional chemotherapeutic and radiotherapeutic agents are toxic to cancer cells by increasing ROS production [18]. Therefore, increasing production of ROS may be an important strategy for cancer therapies.

The endoplasmic reticulum (ER) is a crucial organelle in protein folding, modification, and secretion. A variety of toxic conditions such as hypoxia, failure of protein synthesis, protein misfolding, and Ca^2+^ overload result in ER stress-related events [19,20]. When ER stress occurs, unfolded proteins accumulate and cause the unfolded protein response (UPR) [21]. UPR initiates a signal transduction cascade that is regulated by three conserved ER transmembrane proteins that serve as sensors of ER stress: inositol-requiring kinase 1 (IRE1), protein kinase RNA-like endoplasmic reticulum kinase (PERK) and activating transcription factor 6 (ATF6) [22]. Moreover, UPR induces the expression of ER-resident chaperones, such as glucose-regulated protein (GRP) 78 and GRP94 [23]. A large body of evidence has shown that ER stress has a pivotal role in apoptosis. ER stress is thought to activate mitochondria-dependent cell death pathways [24,25] that utilize members of the Bcl-2 family including Bax and Bak [21]. ER stress also leads to release of Ca^2+^ from the ER and this Ca^2+^ subsequently activates calpains, which could induce apoptosis by promoting the cleavage and activation of apoptosis-related caspases [26]. Activation of the mitochondria-dependent cell death pathway is also accompanied by the accumulation of ROS that can be sequentially converted into toxic ROS, such as hydrogen peroxide, which can independently induce cell death [27,28].

Here, we investigate the anti-cancer activity of Nimbolide. Our data indicate that Nimbolide induces apoptosis and reduces migration of human osteosarcoma cells.

## 2. Results

### 2.1. Nimbolide Induces Apoptosis in Human Osteosarcoma Cells

To investigate whether Nimbolide could induce cell death in human osteosarcoma cells, we first examined the effect of Nimbolide on cell survival by using an MTT assay. Treatment of cells with Nimbolide reduced viability of osteosarcoma cells (MG63, U2OS and HOS cells) but not of non-cancerous osteoblast cells (hFOB 1.19; Figure 1B). The anti-cancer activities of Nimbolide were further assessed with a colony formation assay, and the results indicate that treatment of osteosarcoma cell lines with Nimbolide reduces colony formation in a dose-dependent manner (Figure 1C). To determine whether Nimbolide induces cell death through an apoptotic mechanism, we used DAPI staining and a DNA ladder assay. Nimbolide treatment dramatically increased the amount of condensed and degraded chromatin (Figure 1D,E). We also confirmed that Nimbolide induces apoptosis as shown not only by an increase in the percentages of cells in the sub G1 phase and double-labeled with Annexin V and propidium iodide (PI), but also by a TUNEL assay (Figure 1F–H). These results indicate that Nimbolide induces apoptosis in osteosarcoma cells.

### 2.2. ROS and Mitochondrial Dysfunction Are Involved in Nimbolide-Induced Apoptosis in Human Osteosarcoma Cells

**Figure 2:**
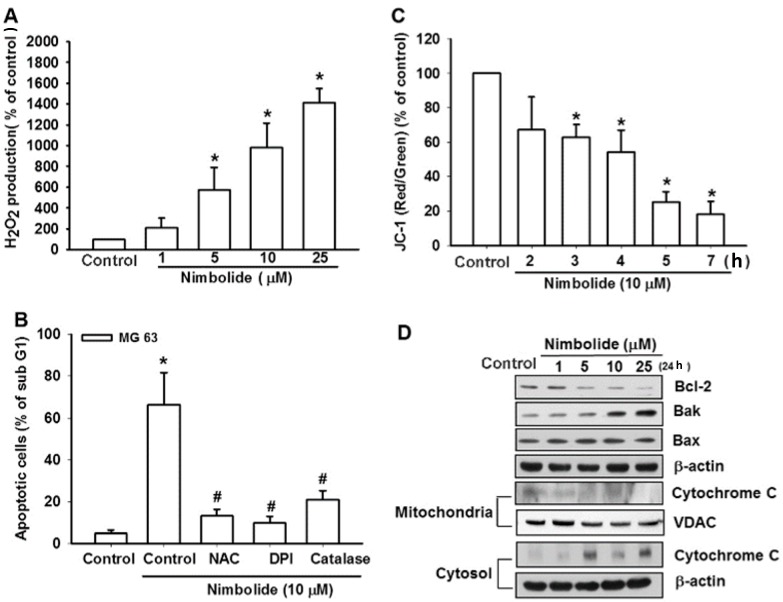
Nimbolide induces the generation of ROS and mitochondrial dysfunction in human osteosarcoma cells. (**A**) MG63 cells were treated with control solution or various concentrations of Nimbolide for 24 h. The concentration of H_2_O_2_ as a measure of ROS production was examined by using flow cytometry; (**B**) MG63 cells were pretreated for 30 min with *N*-acetylcysteine (NAC), diphenyleneiodonium chloride (DPI), and catalase followed by treatment with Nimbolide (10 μM) for 24 h. The percentage of apoptotic cells was then analyzed by flow cytometric analysis of PI-stained cells; (**C**) MG63 cells were treated as described in (**A**). The mitochondrial membrane potential was examined by using flow cytometry and JC-1 staining; (**D**) MG63 cells were treated with control solution or various concentrations of Nimbolide for 24 h. The expression levels of Bcl-2, Bak, Bax, mitochondrial cytochrome c, and cytosolic cytochrome c were examined by using western blot analysis; actin and voltage-dependent anion channels (VDAC) were used as internal controls. The data are expressed as the mean ± SEM. * *p* < 0.05 compared with controls. **^#^** *p* < 0.05 compared with the Nimbolide treated groups.

The results of previous studies indicate that the generation of ROS plays a pivotal role in apoptosis and that ROS can function as anti-cancer agents [29,30]. Therefore, we investigated whether the accumulation of ROS is involved in Nimbolide-induced cell death. FACS analysis indicates that treatment of osteosarcoma cells with Nimbolide induces the accumulation of H_2_O_2_ (Figure 2A). Pretreatment of cells with the ROS scavenger *N*-acetyl cysteine (NAC), the NADPH oxidase inhibitor diphenyleneiodonium chloride (DPI), and catalase (an H_2_O_2_ scavenging enzyme) reduced Nimbolide-induced apoptosis (Figure 2B). ROS production and accumulation in mitochondria have previously been shown to decrease the mitochondrial membrane potential (MMP) and initiate mitochondrial apoptosis [31]. Our data show that treatment of osteosarcoma cells with Nimbolide also decreases the MMP (Figure 2C) as well as alters the expression of members of the Bcl-2 family to increase the ratio of pro-apoptotic to anti-apoptotic Bcl-2 proteins (Figure 2D). These results demonstrate that Nimbolide induces apoptosis in osteosarcoma cells via ROS and mitochondrial apoptosis.

### 2.3. Nimbolide Induces ER Stress in Human Osteosarcoma Cells

**Figure 3:**
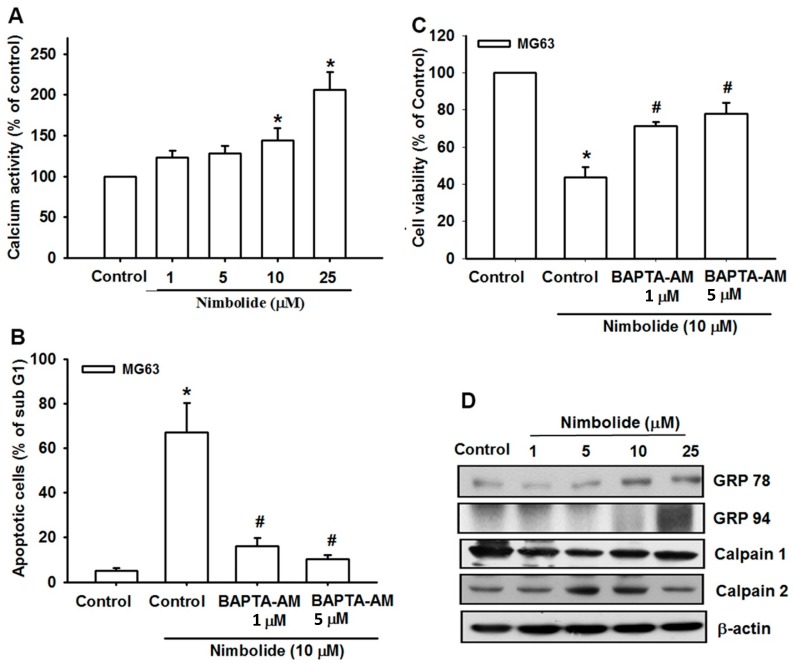
Nimbolide induces Ca^2+^ release and ER stress in human osteosarcoma cells. (**A**) MG63 cells were treated with control solution or various concentrations of Nimbolide for 24 h. Ca^2+^ release was examined by using flow cytometry; (**B**) MG63 cells were pretreated for 30 min with BAPTA-AM (1 or 5 μM) followed by treatment with Nimbolide (10 μM) for 24 h. The percentage of apoptotic cells was then analyzed by flow cytometric analysis of PI-stained cells; (**C**) MG63 cells were treated as described in (**B**), then cell viability was examined by using an MTT assay; (**D**) MG63 cells were treated with control solution or various concentrations of Nimbolide for 24 h. The expression levels of GRP78, GRP94, calpain 1, and calpain 2 were examined by using western blot analysis; actin was used as an internal control. The data are expressed as the mean ± SEM. * *p* < 0.05 compared with controls. **^#^** *p* < 0.05 compared with the Nimbolide treated groups.

The accumulation of oxidative stress in the ER frequently results in initiation of the UPR [32]. Therefore, we investigated the effects of Nimbolide treatment on ER stress. First, we accessed calcium release, which is the link between ER stress and mitochondrial dysfunction [33]. Our results indicate that Nimbolide increases the accumulation of intracellular calcium (Figure 3A). Moreover, pretreatment of osteosarcoma cells with BAPTA-AM, a cell-permeant calcium chelator, reduced apoptosis and increased viability in cells treated with Nimbolide (Figure 3B,C). ER stress is generally characterized by up-regulation of GRP78, GRP94, and calpains 1 and 2, and the expression of all but calpain 1 were increased after Nimbolide treatment (Figure 3D). These results reveal that Nimbolide induces the ER stress-related apoptotic pathway in osteosarcoma cells.

### 2.4. Nimbolide Induces the Activation of Caspases in Human Osteosarcoma Cells

**Figure 4:**
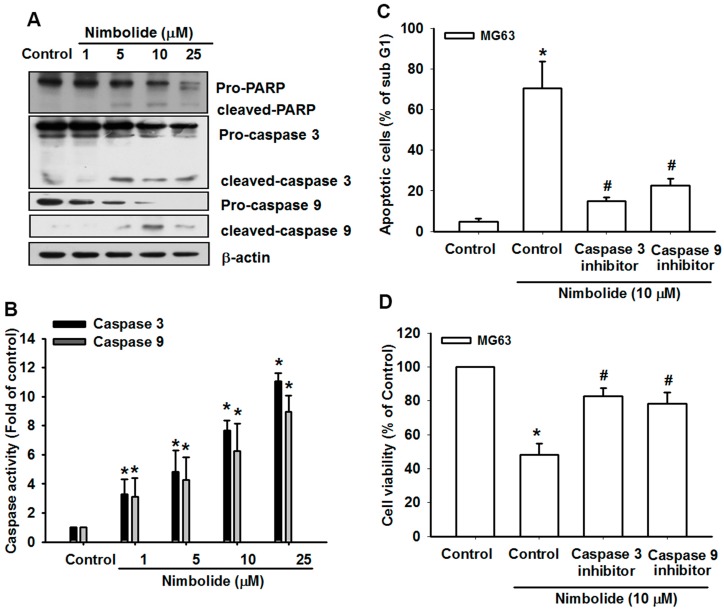
Nimbolide induces the activation of caspases in human osteosarcoma cells. (**A**) MG63 cells were treated with control solution or various concentrations of Nimbolide for 24 h. The expression levels of PARP, caspase-3, and caspase-9 were examined by using western blot analysis; (**B**) MG63 cells were treated as described in (**A**). The activities of caspase-3 and caspase-9 were examined by using a caspase ELISA kit; (**C**,**D**) MG63 cells were pretreated for 30 min with inhibitors of caspase-3 and caspase-9, followed by treatment with Nimbolide (10 μM) for 24 h. The percentages of apoptotic cells were then analyzed by flow cytometric analysis of PI-stained cells (**C**). Cell viability was examined by using an MTT assay (**D**). The data are expressed as the mean ± SEM. * *p* < 0.05 compared with controls. **^#^** *p* < 0.05 compared with the Nimbolide treated groups.

The results presented above suggest that Nimbolide triggers accumulation of ROS, ER stress, and mitochondrial dysfunction in osteosarcoma cells. However, the apoptotic signaling pathway is complex, and the canonical pathway usually involves activated caspases. We determined that treatment with Nimbolide activates caspase-3, caspase-9, and PARP (a downstream effector of caspase-3) (Figure 4A,B). In addition, pretreatment with inhibitors of both caspases abolished Nimbolide-induced cell death and increased viability (Figure 4C,D). These results confirm that caspases are involved in Nimbolide-induced apoptosis.

### 2.5. Nimbolide Reduces Cell Migration by Decreasing the Expression of Integrin αvβ5 in Human Osteosarcoma Cells

**Figure 5:**
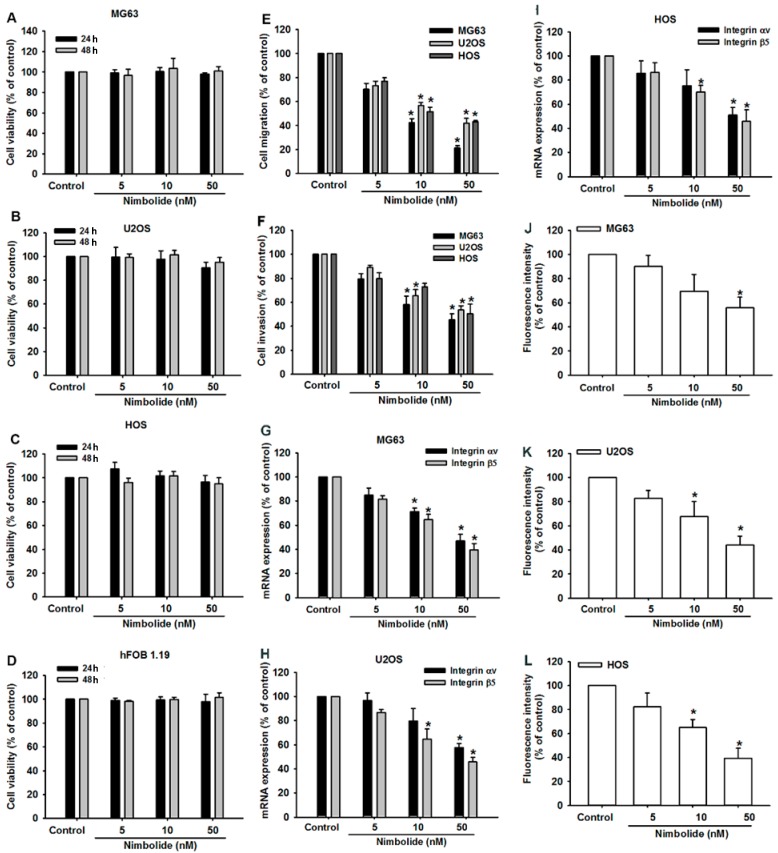
Nimbolide inhibits cell migration of human osteosarcoma cells by modulating the expression of integrin αvβ5. (**A**–**D**) Osteosarcoma cells (MG63, U2OS, and HOS) and osteoblast cells (hFOB 1.19) were incubated with control solution or various concentrations of Nimbolide for 1 or 2 days, then cell viability was examined by using an MTT assay; (**E**,**F**) Osteosarcoma cells (MG63, U2OS, and HOS) were incubated with control solution or various concentrations of Nimbolide for 24 h, then migration and invasion were measured *in vitro* using Transwells; (**G**–**I**) Osteosarcoma cells (MG63, U2OS, and HOS) were incubated with control solution or various concentrations of Nimbolide for 24 h; The expression of *integrin αv* and *β5* mRNAs was examined by qPCR (**J**–**L**). The expression of integrin αvβ5 protein was examined by flow cytometric analysis. The data are expressed as the mean ± SEM. * *p* < 0.05 compared with controls.

Tumor metastasis is the cause of death for many patients with cancer. A critical step in tumor metastasis is migration and invasion of cancerous cells to nearby tissues [34]. Therefore, we accessed the effects of Nimbolide on the cell migration phenotype of osteosarcoma cells. We found that low doses of Nimbolide inhibited cell migration and invasion but did not affect cell viability (Figure 5A–F and Figure S1). Next, we investigated which component of the migration machinery was inhibited by Nimbolide. Integrins have been proposed as pivotal proteins that participate in tumor cell migration, invasion, and metastasis [35,36,37], and it has been proposed that integrin αvβ5 plays a crucial role in the metastasis of OS [38]. As we expected, Nimbolide treatment decreased the expression of *integrin αvβ5* mRNA and protein in osteosarcoma cell lines (Figure 5G–L). These results reveal that Nimbolide may regulate migration of osteosarcoma cells through integrin αvβ5. Future research will confirm the role of integrin αvβ5 in Nimbolide regulation of migration of osteosarcoma cells.

### 2.6. The PI3K/Akt and NF-κB Signaling Pathway Mediates Nimbolide Inhibition of Cell Migration in Osteosarcoma Cells

**Figure 6:**
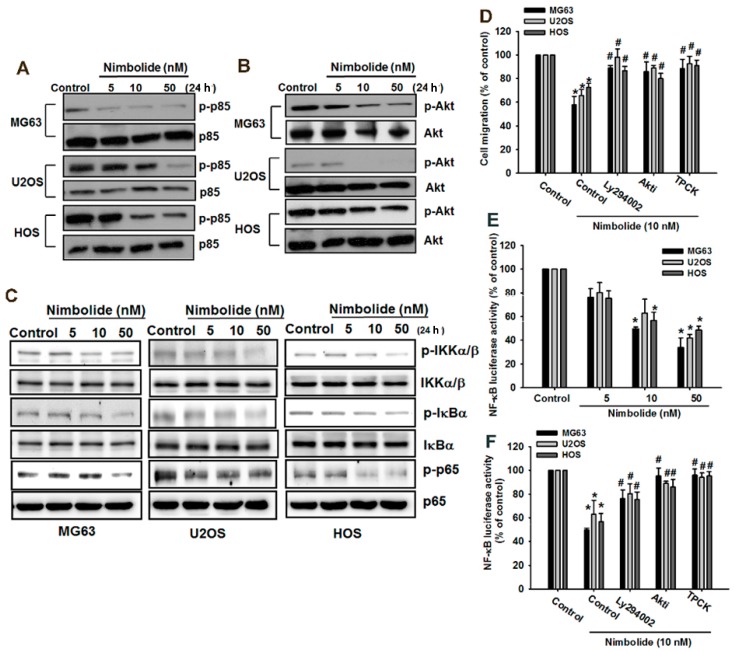
PI3K/Akt and NF-κB pathways function downstream of Nimbolide to inhibit cell migration in human osteosarcoma cells. (**A**–**C**) Osteosarcoma cells (MG63, U2OS, and HOS) were treated with control solution or various concentrations of Nimbolide for 24 h. The expression levels of phosphorylated (p)-p85, p85, p-Akt, Akt, p-IκBα, IκBα, p-IKKα/β, IKKα/β, p-p65 and p65 were examined by using western blot analysis; (**D**) Osteosarcoma cells (MG63, U2OS, and HOS) were pretreated with control solution or various concentrations of Nimbolide for 24 h; (**E**) Osteosarcoma cells (MG63, U2OS, and HOS) were transfected with an NF-κB promoter reporter plasmid for 24 h. The cells were then treated as described in (**A**) and luciferase activity was measured; (**F**) Osteosarcoma cells (MG63, U2OS, and HOS) were pretreated with PI3K inhibitors (LY294002 (5 μM) and Wortmannin (0.5 μM)), Akt inhibitor (Akti (5 μM)), or NF-κB inhibitors (TPCK (5 μM)) for 30 min before exposure to AREG. NF-κB luciferase activity was measured, and the results were normalized to the β-galactosidase activity. The data are expressed as the mean ± SEM. * *p* < 0.05 compared with controls. **^#^** *p* < 0.05 compared with the Nimbolide treated groups.

To investigate the mechanism by which Nimbolide inhibits cell migration in osteosarcoma cells, we assessed which signaling pathways it regulates. Previous studies have indicated that the PI3K/Akt and NF-κB signaling pathway is regulated by Nimbolide [5,39]. We examined the expression and phosphorylation status of Akt and the p85 subunit of PI3K in osteosarcoma cells, and found decreased phosphorylation of both proteins (Figure 6A,B). NF-κB transcription factors are localized to the cytoplasm, where they bind to the inhibitory protein IκB, which inhibits NF-κB binding and prevents nuclear translocation. Upon stimulation of cells, IκB proteins promote rapid phosphorylation by the multisubunit IKK complex. Phosphorylation then targets IκB for ubiquitination and subsequent degradation by the 26S proteasome. Phosphorylation of IKKα/β, IκBα and the p65 subunit of NF-κB were all decreased by Nimbolide treatment (Figure 6C). Pretreatment with inhibitors of PI3K (Ly294002), Akt (Akti), and NF-κB (TPCK) blocked the effects of Nimbolide in a migration assay (Figure 6D). Moreover, we used a κB-luciferase activity assay to confirm that NF-κB activity was decreased by treatment of cells with Nimbolide (Figure 6E). Finally, we confirmed that pretreatment with Ly294002, Akti, and TPCK restored activity of the NF-κB luciferase reporter (Figure 6F). These results illustrate the pivotal role of the PI3K/Akt and NF-κB signaling cascade in Nimbolide function.

## 3. Discussion

OS is an infrequent malignancy that always occurs in the adolescent. The lung is the most common site of metastasis for OS. Accordingly, it is critical that we develop novel drugs that will reduce OS metastasis and improve prognosis. Tumor metastasis is a complicated process that is mediated by a large number of cellular components. Therefore, cocktail therapies or multi-functional drugs would be more ideal candidates than are drugs that inhibit a single target. In this study, we found that the anti-tumoral effects of Nimbolide target multiple cellular processes of osteosarcoma cells. Nimbolide activated the apoptotic signaling pathway through eliciting production of ROS, ER stress, mitochondrial dysfunction, and finally, caspase activation. The details of the signaling cascade that mediates Nimbolide-induced apoptosis will be examined in the future. We also determined that Nimbolide inhibits cell migration, an activity critical to metastasis, through reducing the expression of integrin αvβ5. In addition, we found that *integrin αvβ5* expression is regulated by the PI3K/Akt and NF-κB signaling cascades. In conclusion, these results indicate that Nimbolide could serve as an anti-tumor drug for OS via its multifunctional effects.

Cancer progression is possible only if the tumor cells evade apoptosis. This is accomplished by modulating the balance between pro- and anti-apoptotic proteins and down-regulation of death receptors [40]. Triggering apoptosis in cancer cells has been recognized as an effective strategy to inhibit cancer progression [41]. A large number of studies have shown that the triggering of apoptosis by ROS has an anti-tumoral effect in many cancers [42]. Apoptosis can be induced by ROS through many different mechanisms, such as cell cycle arrest, and the activation of several signaling pathways including caspase-dependent, caspase-independent, death receptor, mitogen-activated protein kinase, extracellular signal-regulated kinase, and phosphoinositide 3-kinase pathways [43,44,45,46]. Furthermore, levels of ROS are lower and the levels of antioxidants are higher in resistant cancer cells than in non-resistant cancer cells [47]. Therefore, in both types of cancer cells, ROS plays vital roles as chemotherapeutic agents. Several studies have reported that Nimbolide induces apoptosis via ROS production in colon cancer cells and human choriocarcinoma cells [6,8]. The ER is responsible for protein folding and synthesis of secretory proteins. Accumulation of misfolded proteins, hypoxia, Ca^2+^ depletion, nutrient starvation, oxidative stress, and other metabolic dysregulation can all cause ER stress [48]. ER stress induces apoptosis via activation of IRE1, phosphorylation of eIF2a, and release of Ca^2+^ from the ER [49]. The increase in cytosolic calcium can activate calpains, which activate caspase-dependent pathways [50]. Calpain belongs to the calcium-activated non-lysosomal cysteine protease family, which comprises 14 members in mammals. Calpain 1 (μ-calpain) and calpain 2 (m-calpain) are the best-characterized isoforms of calpain [51]. Examining the effect of Nimbolide treatment on calpain expression, we observed an acute increase in the level of calpain 2 protein after cells were treated with 5 and 10 μM Nimbolide for 24 h. We propose that increase in calpain 2 was not due to increased transcription and the levels of calpain 2 protein are increased due to decreased degradation. Therefore, in cells exposed to a higher dose of Nimbolide, we observed the opposite effect. The rapid increase in calpain 2 expression after Nimbolide treatment suggests that this protein is important in cell apoptosis and is directly regulated by Nimbolide.

Few studies have examined the potential of Nimbolide for treatment of OS. The results presented in this report are the first to show that Nimbolide induces apoptosis in osteosarcoma cells through ROS, ER stress, mitochondrial dysfunction, and caspase activation. Moreover, Nimbolide has been reported to regulate expression of the Bcl-2 family of proteins, disrupt the MMP, activate caspases, and trigger apoptosis in breast cancer [52]. Our findings in osteosarcoma cells are in agreement with those of previous studies. Interestingly, we found that Nimbolide induces ER stress in osteosarcoma cells. This effect is novel and has not yet been documented for other types of cancer.

Cancer metastasis occurs in multiple steps by which cancer cells migrate from the primary site and form a secondary tumor at a distant site [53]. The expression and tumor-promoting functions of integrins have been well studied [36]. The present study showed that Nimbolide inhibited migration of osteosarcoma cells by decreasing the expression of integrin αvβ5. Although the role of Nimbolide in inhibiting cell migration in cancer has been characterized, the mechanisms underlying this regulation remain largely unknown. Here, we have provided novel insight regarding the inhibitory effects of Nimbolide. Previous studies have shown that Nimbolide modulates cell signaling cascades including the PI3K/Akt and NF-κB pathway [5,39]. We have also found that these pathways were inhibited by Nimbolide treatment in osteosarcoma cells. Moreover, our data indicate that Nimbolide regulates the expression of integrin αvβ5 via the PI3K/Akt and NF-κB pathway. Future research should clarify if additional signaling cascades are modulated by Nimbolide.

A better way to halt tumor progression may be to use a combination of several drugs or a drug with multiple targets. Previous reviews have discussed the anti-tumoral effects of Nimbolide [54,55]. In this report, we present the first evidence that Nimbolide may be able to prevent the progression of OS by inducing apoptosis and decreasing migration of osteosarcoma cells. Nimbolide has been reported as a potential chemopreventive agent that is able to prevent cancer progression through multiple mechanisms. Whether Nimbolide can suppress the progression of OS by other mechanisms such as inhibiting proliferation and angiogenesis will be investigated in the future.

## 4. Experimental Section

### 4.1. Materials

Nimbolide was obtained from Sigma-Aldrich (St. Louis, MO, USA). Horseradish peroxidase-conjugated anti-mouse and anti-rabbit IgG, and rabbit polyclonal antibodies specific for VDAC, cytochrome c, Bcl-2, Bax, Bak, Bad, Bid, GRP78, GRP94, calpain 1, calpain 2, PI3-kinase (p85), Akt, and NF-κB (p65) were purchased from Santa Cruz Biotechnology (Santa Cruz, CA, USA). Rabbit polyclonal antibodies specific for IKKα/βphosphorylated at serine (Ser)180/181, IκBα phosphorylated at Ser32/36, p65 phosphorylated at Ser536, Akt phosphorylated at Ser473, p85 phosphorylated at tyrosine (Tyr)458, caspase-3, and caspase-9 were purchased from Cell Signaling (Danvers, MA, USA). An NF-κB luciferase plasmid was purchased from Stratagene (La Jolla, CA, USA). A pSV-β-galactosidase vector and luciferase assay kit were purchased from Promega (Madison, WI, USA). All other chemicals were obtained from Sigma-Aldrich.

### 4.2. Cell Culture

The human osteosarcoma cell lines (MG63, U2OS, HOS) and osteoblast cell line (hFOB 1.19) were purchased from the American Type Culture Collection (BCRC; Hsinchu, Taiwan). MG63, U2OS, HOS, and hFOB 1.19 cells were maintained in DMEM, McCoy’s 5A, DMEM, and DMEM/Ham’s F-12 media, respectively. MG63, U2OS, and HOS cells were incubated at 37 °C in an atmosphere of 5% CO_2_ in media supplemented with 20 mM of 4-(2-hydroxyethyl)-1-piperazineethanesulfonic acid (HEPES), 10% heat-inactivated fetal calf serum (FBS), 2 mM glutamine, 100 U/mL penicillin, and 100 μg/mL streptomycin (Invitrogen, Carlsbad, CA, USA). hFOB 1.19 cells were incubated at 34 °C in an atmosphere of 5% CO_2_ in medium supplemented with 10% heat-inactivated FBS and 0.3% G418 (Sigma-Aldrich). All media were changed every 48 h.

### 4.3. Authentication of Cell Lines

The human osteosarcoma cell lines MG63 (BCRC No.60279), U2OS (BCRC No.60187), and HOS (BCRC No.60308), and osteoblast cell line hFOB 1.19 (BCRC No.60357) were obtained from the Biosource Collection and Research Center (BCRC; Hsinchu, Taiwan) in October 2012. The cell lines were tested and authenticated by the BCRC. We purchased the cell lines from the BCRC and passaged it in our laboratory for fewer than 6 months after the cells were thawed.

### 4.4. MTT Assay

Cell viability was determined by using a 3-(4,5-dimethylthiazol-2-yl)-2,5-diphenyltetrazolium bromide (MTT) assay. After treatment with control solution or different concentrations of Nimbolide for 1 or 2 days, the cultured cells were washed with PBS. MTT (0.5 mg/mL) was then added to each well and the mixture was incubated for 2 h at 37 °C. Culture media were then replaced with an equal volume of DMSO to dissolve formazan crystals. After the mixture was shaken at room temperature for 10 min, the absorbance of the solution in each well was determined at 550 nm by using a microplate reader (Bio-Tek, Winooski, VT, USA).

### 4.5. Colony Formation Assay

Cells (1 × 10^3^) were seeded into 6-well plates and cultured for 10 days. Media were replaced every 2 days. After incubation with control solution or different concentrations of Nimbolide for 10 days, colonies were washed with PBS, fixed for 15 min with 4% paraformaldehyde and stained with 0.1% crystal violet for 5 min. The colony forming cells were photographed. After the colonies were washed 3 times with ddH_2_O, acetic acid was added to a final concentration of 33% (*v*/*v*), which was followed by measuring the absorbance at 550 nm with a microplate reader. The colony formation assay was repeated 3 times, each of which was performed in duplicate wells.

### 4.6. DAPI Staining

4′-6-Diamidino-2-phenylindole (DAPI), a DNA-binding fluorescent dye, was used to monitor apoptosis by observing nuclear morphology. After treatment with Nimbolide for 48 h, the cells were washed 3 times with PBS, fixed in a 3.7% formaldehyde solution for 10 min, fixed in 1 mL of methanol, and then stained with DAPI for 10 min. Apoptosis was determined by visual observation of nuclear morphology with fluorescence microscopy.

### 4.7. DNA Ladder Assay

MG63 cells (1 × 10^6^) were treated with control solution or Nimbolide for 48 h, then total DNA was extracted and analyzed with agarose gel electrophoresis (2%). Ethidium bromide staining was used to assess DNA fragmentation as an indicator of apoptosis. Ultraviolet spectroscopy at 302 nm was used to visualize the DNA after electrophoresis.

### 4.8. Western Blot Analysis

The cell lysates were prepared as described previously [56]. Proteins were resolved with SDS-PAGE and transferred to immobilon polyvinylidene difluoride (PVDF) membranes. The membranes were blocked with 4% BSA for 1 h at room temperature and then probed with primary antibodies (1:1000) for 1 h at room temperature. After 3 washes, the membranes were subsequently incubated with peroxidase-conjugated secondary antibodies (1:1000) for 1 h at room temperature. The protein bands were visualized by enhanced chemiluminescence with Kodak X-OMAT LS film (Eastman Kodak, Rochester, NY, USA).

### 4.9. Cytochemical Analysis with Annexin V and Propidium Iodide (PI)

Apoptosis was assessed by using annexin V, a protein that binds to phosphatidylserine residues, which are exposed on the cell surface of apoptotic cells, as previously described [57]. Cells were treated with control solution or Nimbolide for the indicated time intervals. After treatment, the cells were washed twice with PBS, and then resuspended in staining buffer containing 1 μg/mL PI and 0.025 μg/mL FITC-conjugated annexin V. Double-labeling was performed at room temperature for 10 min in the dark, then analyzed immediately by using FACScan and the Cellquest program (BD Biosciences, San Diego, CA, USA).

### 4.10. Cell Cycle Analysis by PI Staining

Quantitative assessment of apoptotic cells was also performed by examining the cell cycle. Cells were collected by centrifugation and the concentration was adjusted to 3 × 10^6^ cells/mL. Chilled ethanol was added to 0.5 mL of cell suspension and the mixture was incubated at 4 °C for 30 min. Ethanol was then removed by centrifugation, and cellular DNA was stained with 100 μg/mL PI (in PBS containing 0.1% Triton-X 100 and 1 mM EDTA) in the presence of an equal volume of DNase-free RNase (200 μg/mL). After staining, the cells were analyzed immediately with a FACScan and the Cellquest program. The extent of apoptosis was determined by measuring the DNA content of cells below the sub G1 peak [58].

### 4.11. Terminal Deoxynucleotidyl Transferase dUTP Nick End Labeling (TUNEL) Assay

Quantitative assessment of apoptotic cells was also performed by using a TUNEL assay (BD ApoAlert™ DNA Fragmentation Assay Kit, BD Biosciences Clontech, Palo Alto, CA, USA), which examines DNA-strand breaks during apoptosis. Briefly, cells were incubated with control solution or Nimbolide for the indicated times. The cells were trypsinized, fixed with 4% paraformaldehyde, and then permeabilized with 0.1% Triton-X 100 in 0.1% sodium citrate. After being washed, the cells were incubated with the reaction mixture for 60 min at 37 °C. The stained cells were then analyzed by using flow cytometry.

### 4.12. Determination of Mitochondrial Membrane Potential (MMP)

The MMP (ΔΨm) was assessed by using the fluorometric probe JC-1 (Calbiochem, CA, USA), a positively charged mitochondria-specific fluorophore that indicates depolarization by a shift in fluorescence emission from green (525 nm) to red (610 nm) [59]. Briefly, the cells were plated in 6-well culture dishes, grown to confluence, and then treated with control solution or Nimbolide. After incubation, the cells were stained with JC-1 (5 μg/mL) for 15 min at 37 °C and then analyzed by FACScan using an argon laser (488 nm). Mitochondrial depolarization, which is specifically indicated by a decrease in the intensity ratio of red to green fluorescence, was analyzed by using the Cellquest program.

### 4.13. Measurement of ROS

Levels of intracellular H_2_O_2_ were assessed by using spectrofluorometry to monitor the oxidation of the probe H_2_DCFDA (Molecular Probes, Waltham, MA, USA). Cells were plated at a density of 5 × 10^5^, allowed to attach overnight, and exposed to control solution or Nimbolide for the specified time intervals. The cells were incubated with H_2_DCFDA (10 μM) for 10 min at 37 °C and the fluorescence intensity in the cells was determined by using flow cytometry.

### 4.14. Detection of Ca^2+^ Concentration

To detect changes in Ca^2+^ levels, 2 × 10^5^ cells were plated in 12-well plates and incubated with control solution or Nimbolide for the specified time intervals. The cells were harvested, washed twice, resuspended in Fluo 3/AM (3 μg/mL), and incubated at 37 °C for 30 min before being analyzed by using flow cytometry.

### 4.15. Measurement of Caspase Activity

The caspase activity assay is based on the ability of the active caspase enzyme to cleave the chromophore p-nitroaniline from a peptide substrate: Ac-DEVD-pNA was the substrate for caspase-3 and Ac-LEHD-pNA was the substrate for caspase-9. The cell lysates were prepared and incubated with antibodies specific for caspase-3 and caspase-9. The immunocomplexes were incubated with the substrate in assay buffer (100 mM NaCl, 50 mM 4-[2-hydroxyethyl]-1-piperazine-ethanesulphonic acid [HEPES], 10 mM dithiothreitol, 1 mM EDTA, 10% glycerol, 0.1% 3-[(3-cholamidopropyl) dimethyl ammonio]-1-propanesulfonate [CHAPS], pH 7.4) for 2 h at 37 °C. The release of p-nitroaniline was monitored at 405 nm with a microplate reader. The results are presented as the percent change of activity between the test sample and the untreated control.

### 4.16. In Vitro Cell Migration and Invasion Assays

The migration assay was performed with Transwell inserts (Costar, NY, USA; 8-mm pore size) in 24-well dishes. Approximately 1 × 10^4^ cells in 200 μL of serum-free medium were placed in the upper chamber, and 300 μL of the same medium containing control solution or different concentrations of Nimbolide were placed in the lower chamber. The cells were incubated for 24 h, then fixed in 3.7% formaldehyde for 5 min and stained with 0.05% crystal violet in PBS for 15 min. The cells on the upper side of the filters were removed and the filters were washed with PBS. The cells on the underside of the filters were examined and counted by using light microscopy. Each experiment was performed in triplicate and repeated at least 3 times. The number of migrated cells in each experiment was corrected for the effects of proliferation by using a cell viability assay (corrected invading cell number = counted invading cell number/percentage of viable cells) [60]. For the invasion assay, Transwell inserts were coated with BD Matrigel (BD Biosciences) before use. The experimental protocol for the invasion assay was as described above for the cell migration assay.

### 4.17. Reporter Gene Assays

Cells were co-transfected with an NF-κB reporter plasmid (0.7 µg) and a p-SV-β-galactosidase plasmid (0.3 µg) by using Lipofectamine 2000 according to the manufacturer’s recommendations. After transfection for 24 h, the cells were treated with control solution or Nimbolide for 24 h. Cell extracts were then prepared, and the activities of luciferase and β-galactosidase were measured.

### 4.18. Statistical Analyses

The values reported are means ± SEM. Statistical analyses of differences between 2 samples were performed by using the Student’s *t-*test. Statistical comparisons of more than 2 groups were performed by using 1-way analysis of variance (ANOVA) with Bonferroni’s *post-hoc* test. In all cases, *p* < 0.05 was considered significant.

## 5. Conclusions

Our data indicate that Nimbolide induces apoptosis and inhibits cell migration in human osteosarcoma cells. Nimbolide-induced cell death is mediated by increasing levels of ROS, mitochondrial dysfunction, ER stress, and Ca^2+^ release, all of which subsequently lead to increased activity of calpain, caspase-3, and caspase-9. Furthermore, we have also shown that Nimbolide inhibits cell migration by modulating the expression of integrin αvβ5, which is regulated by the PI3K/Akt and NF-κB signaling cascade. We hope that our proposed working model for the molecular basis of Nimbolide function will provide valuable insights for the development of an effective chemotherapy for OS by targeting the appropriate signal transduction proteins.

## Supplementary Materials

Supplementary materials can be found at http://www.mdpi.com/1422-0067/16/10/23405/s1.
