# Supplementary material for: Nimbolide Induces ROS-Regulated Apoptosis and Inhibits Cell Migration in Osteosarcoma
Source: Int J Mol Sci. 2015 Sep 29;16(10):23405–24. doi: 10.3390/ijms161023405 (PMC4632706; doi:10.3390/ijms161023405)
Supplement: Supplementary file 1 [file ijms-16-23405-s001.pdf]

Supplementary Information

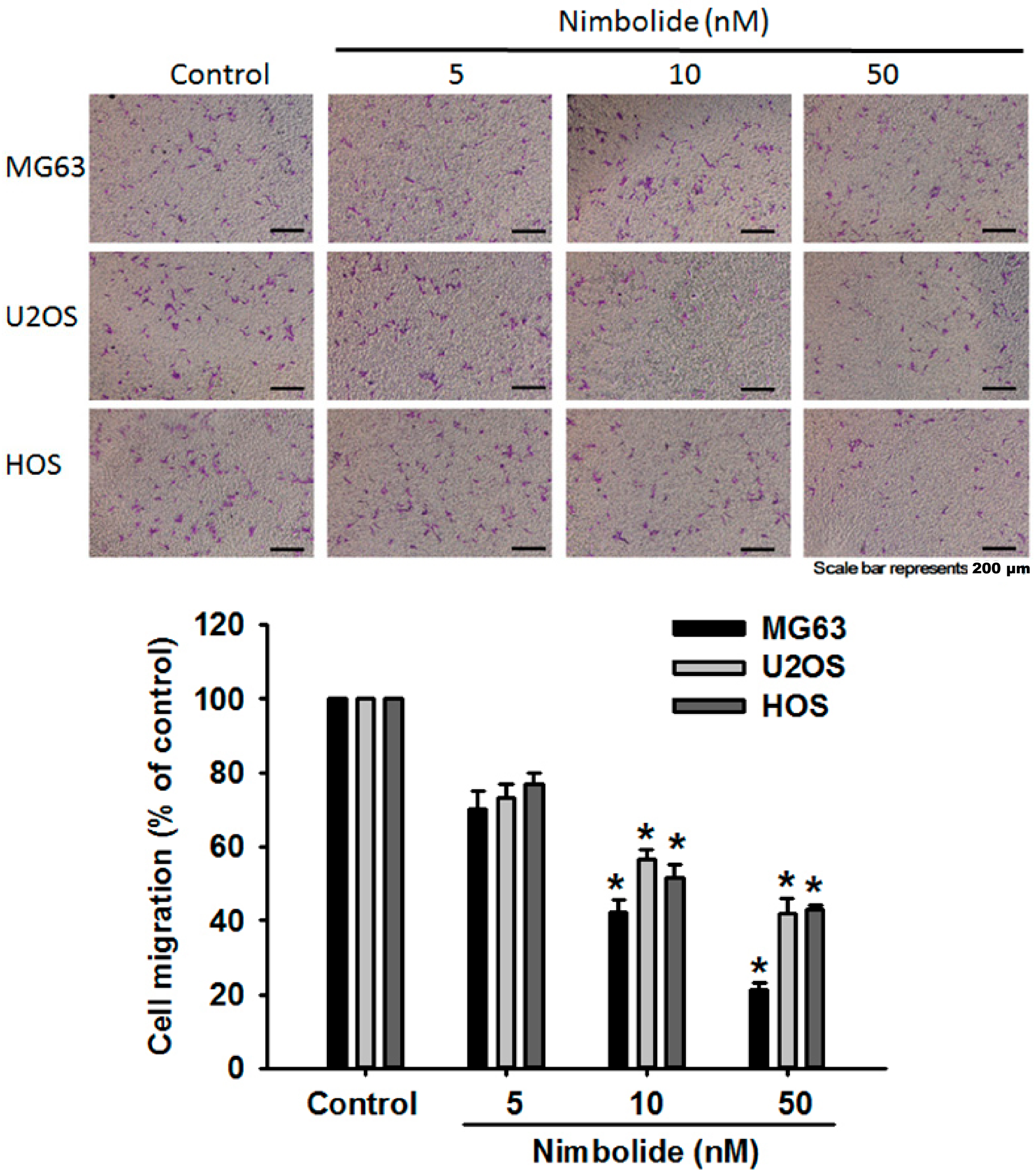

Figure S1. Nimbolide inhibits cell migration of human osteosarcoma cells. Osteosarcoma cells (MG63, U2OS, and HOS) were incubated with control solution or various concentrations of Nimbolide for 24 h, then migration and invasion were measured *in vitro* by using Transwells. Scale bar: 200 μm.
